# Efficacy of Outside‐In Hip Arthroscopy without Traction in the Treatment of Hip Synovial Osteochondromatosis

**DOI:** 10.1111/os.14258

**Published:** 2024-10-01

**Authors:** Weigang Wu, Meng Liu, Chenhe Zhou, Huajie Mao, Huiguo Wu, Zhiqiang Wu, Chiyuan Ma

**Affiliations:** ^1^ Department of Orthopedics Surgery, The Second Affiliated Hospital Zhejiang University School of Medicine Hangzhou PR China; ^2^ Orthopedics Research Institute of Zhejiang University Hangzhou PR China; ^3^ Key Laboratory of Motor System Disease Research and Precision Therapy of Zhejiang Province Hangzhou PR China; ^4^ People's Hospital of Changxing County Huzhou PR China; ^5^ Suichang County People's Hospital in Zhejiang Province Lishui PR China

**Keywords:** Hip Synovial Osteochondromatosis, Neurovascular Injury, No Traction, Outside‐In Hip Arthroscopy, Peripheral Compartment

## Abstract

Arthroscopic treatments of hip synovial osteochondromatosis are mostly performed under traction, resulting in neurovascular injury or iatrogenic damage to the labrum or cartilage. This study aimed to assess the effectiveness of outside‐in hip arthroscopy without traction in treating hip synovial osteochondromatosis. This retrospective study was conducted on a series of patients with hip synovial osteochondromatosis treated using outside‐in hip arthroscopy without traction in our hospital between 2018 and 2020. Plain radiography and magnetic resonance imaging (MRI) scans were obtained. The Harris hip score (HHS), hip range of motion (ROM), and visual analog scale (VAS) scores were analyzed. The preoperative scores and last follow‐up scores were compared with a paired‐sample *t* test. The complications and recurrence postsurgery were recorded. This study included five patients (three male and two female) with an average age of 41 years (range 28–54 years). The mean follow‐up time was 25.2 months (range 18–36 months). All patients experienced groin pain relief and improved ROM. The mean VAS score was significantly lower postoperatively (0.4 ± 0.5) than preoperatively (3.2 ± 0.8) (*p* < 0.001). The mean HHS improved from 58.6 ± 12.7 (range 43–73) to 89.8 ± 5.26 (range 81–95) (*p* < 0.001). No major complications, including infection, perineal numbness and swelling, neurotrosis, thromboembolism, or severe persistent pain, were reported. Synovial osteochondromatosis recurred in one patient after 2 years of follow‐up without any obvious symptoms such as hip pain or joint locking. Therefore, no further treatment was necessary. This study showed that outside‐in hip arthroscopy without traction might be a viable option for treating hip synovial osteochondromatosis, effectively and safely relieving symptoms with minimal complications, especially in patients without lesions in the central compartment.

## Graphical abstract

(A) Outside‐in hip arthroscopy in the supine position on a regular operation table without traction. (B) Fluoroscopic image of outside‐in arthroscopic access to the hip.

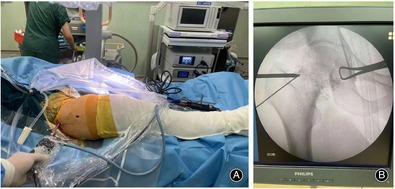

## Introduction

Hip synovial osteochondromatosis results in multiple intra‐articular loose bodies and synovial hyperplasia, which is a rare benign metaplasia of the synovium.^1^, ^2^, ^3^ Symptoms typically include pain and swelling, joint deviation, and limited hip motion.^4^, ^5^ The loose bodies can be removed surgically using either an open^6^, ^7^, ^8^ or an arthroscopic approach.^9^, ^10^, ^11^ The arthroscopic treatment of this condition has become increasingly popular in recent years due to its minimally invasive nature and associated advantages.^12^, ^13^, ^14^ Most arthroscopic treatments are performed under traction, resulting in neurovascular injury or iatrogenic damage to the labrum or cartilage.^15^, ^16^ Complete hip arthroscopy typically requires traction, especially for assessing the central compartment. However, the accumulation of loose bodies in the peripheral compartment is common, where they are easily visible without traction. Hip arthroscopy without traction can avoid the risk of neurovascular injury or iatrogenic damage to the labrum or cartilage. Furthermore, extracapsular outside‐in hip arthroscopy, introduced at the end of the 20th century, has significantly enhanced arthroscopic visualization.^17^ To the best of our knowledge, few studies have reported the application of outside‐in hip arthroscopy without traction in treating hip synovial osteochondromatosis. This study reported an outside‐in hip arthroscopy procedure without traction for a series of cases and evaluated its efficacy.

The primary objectives of this study were to (i) describe the outside‐in hip arthroscopy without traction in the treatment of hip synovial osteochondromatosis and (ii) analyze the medical records of those cases to evaluate the efficacy of this technique.

## Methods

### Patient Selection

**FIGURE 1:**
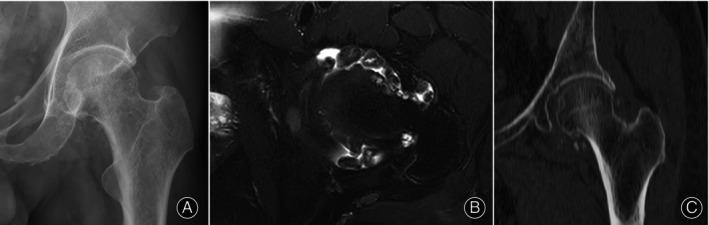
Radiographic appearance of hip synovial osteochondromatosis (left hip, PT‐5): (A) x‐ray, (B) magnetic resonance (MR) T2‐weighted image, and (C) computed tomography (CT) coronal reconstruction image.

We retrospectively reviewed the cases of all patients with primary hip synovial osteochondromatosis treated with synovectomy and removal of loose bodies using outside‐in hip arthroscopy without traction in our hospital between 2018 and 2020. It was approved and performed in agreement with the ethical standards of our hospital research committee (No. 2024‐0824). Informed consent was obtained. This arthroscopy was indicated for patients with intra‐articular bodies mainly in the peripheral compartment. Furthermore, this arthroscopy was not indicated for patients with symptoms requiring treatment in the central compartment under traction. These patients were diagnosed based on their radiographic appearance (Figure 1), which included numerous intra‐articular radiopaque bodies in the peripheral compartment but with no obvious loose bodies, cartilage injury in the central compartment, or femoroacetabular impingement (FAI) syndrome.

The inclusion criteria were as follows: (1) patients who underwent synovectomy and removal of loose bodies using outside‐in hip arthroscopy without traction for primary hip synovial osteochondromatosis and (2) histological results confirming the diagnosis of synovial osteochondromatosis. The exclusion criteria were as follows: (1) a follow‐up period of less than 1 year and (2) cases with incomplete follow‐up data.

### Surgical Technique

**FIGURE 2:**
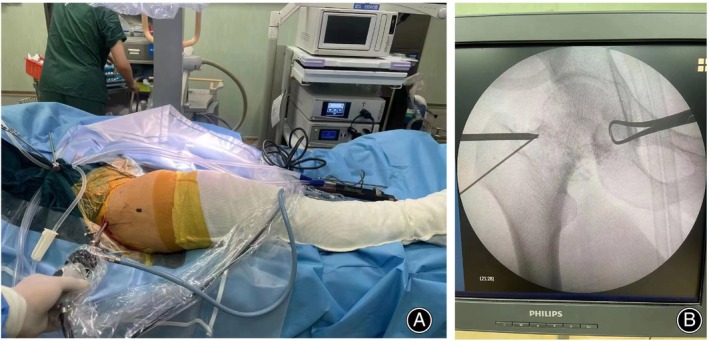
(A) Outside‐in hip arthroscopy in the supine position on a regular operative table without traction. (B) Fluoroscopic image of outside‐in arthroscopic access to the hip.

The patients were placed on a regular operative table without traction after general anesthesia. Preoperative limb preparation was then completed in a sterile manner, and bony landmarks and arthroscopic portals were marked on the skin. Hip motion was used to enhance arthroscopic vision because the leg was placed on the table or held by the assistant without fixation. The legs were placed in slight internal rotation before the portal was created to accommodate the femoral version. The fluoroscopy confirmed the extra‐articular placement of the anterolateral portal using a long needle. Following the threading of a nitinol guidewire, a trocar was used to establish a primary viewing portal for the anterosuperior compartment of the joint capsule. An arthroscope was inserted, and irrigation was used to clear the view. A long needle was then inserted into the mid‐anterior portal and triangulated into the extracapsular space under arthroscopy. Then, a nitinol wire was inserted into the joint capsule, and a trocar was placed for establishing the working portal (Figure 2).

**FIGURE 3:**
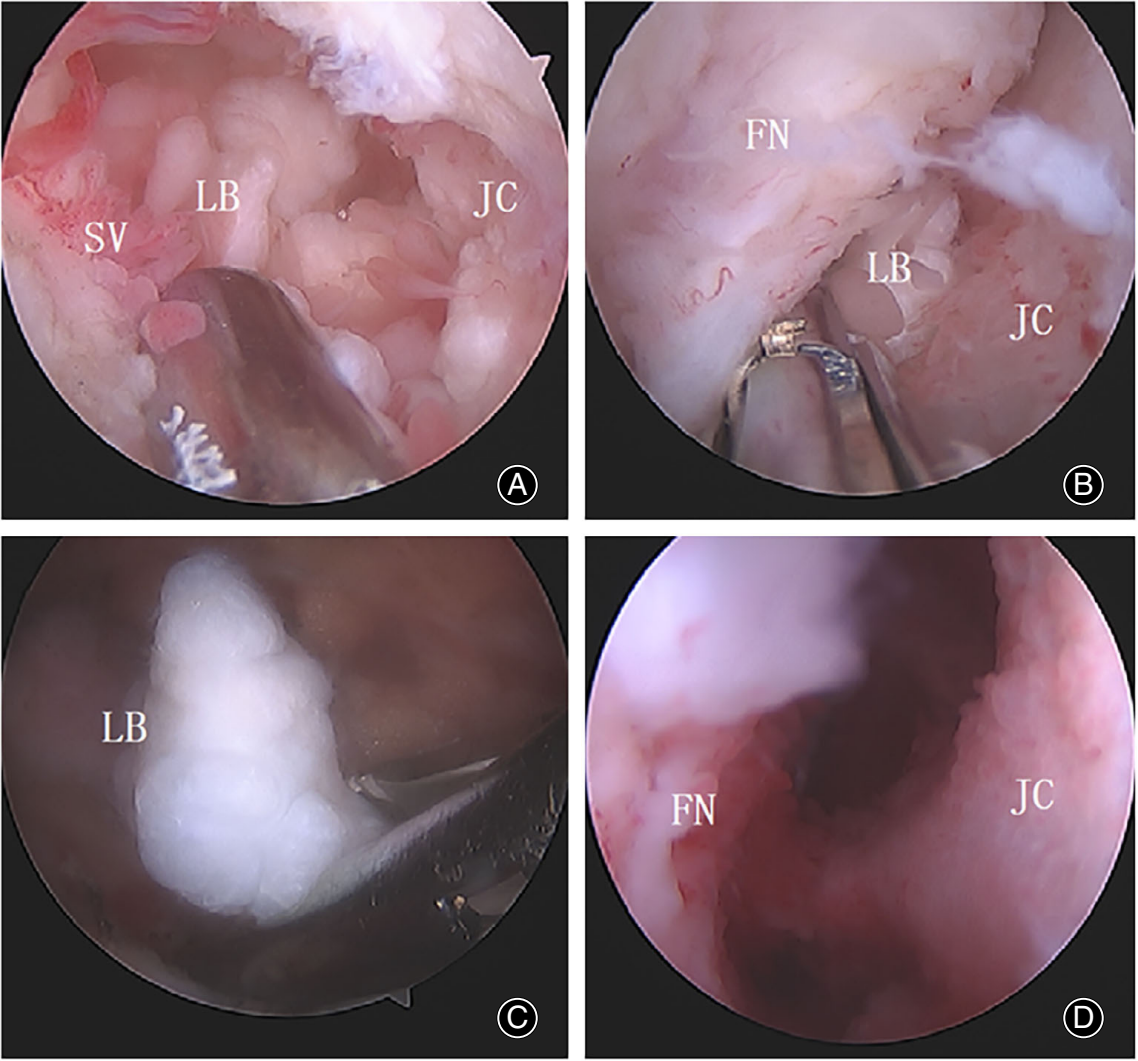
(A) Arthroscopic view of loose bodies in the peripheral compartment. (B,C) Arthroscopic removal of a loose body using a tissue grasper. (D) Arthroscopic view of the posteroinferior region after arthroscopic loose body removal and synovectomy. LB, loose body; SV, synovium; FN, femoral neck; JC, joint capsule.

**FIGURE 4:**
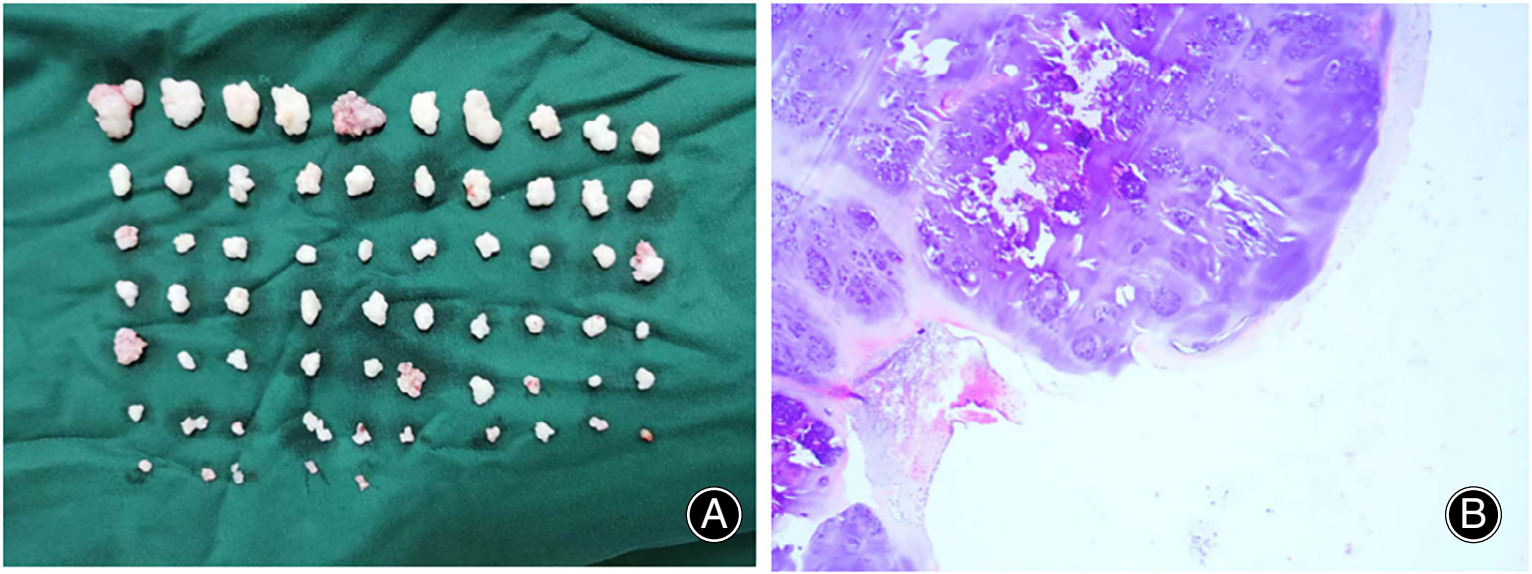
(A) Multiple loose bodies removed under arthroscopy. (B) Histological appearance with irregular islands of cartilage (hematoxylin and eosin stain, image magnification ×50).

**FIGURE 5:**
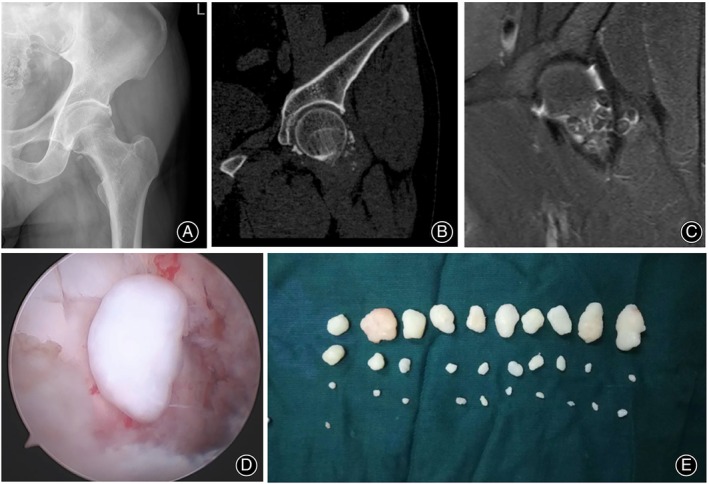
The size of loss bodies varies greatly in PT‐1. Radiographic appearance: (A) x‐ray, (B) computed tomography (CT) coronal reconstruction image, and (C) magnetic resonance (MR) T2‐weighted image. (D) Arthroscopic view. (E) Multiple loose bodies in different sizes.

A shaver was then used to clean the view and remove soft tissues in the extracapsular space of the hip. An anterior capsulotomy was performed to open the joint capsule under direct visualization from the extracapsular space. The peripheral compartment was examined in a diagnostic round trip starting from the anterior surface of the femoral neck. Different areas of the peripheral compartment of the hip could be viewed, especially the posteroinferior and anteroinferior regions where the loose bodies were primarily located, under slow rotation and sliding of the arthroscope over the femoral neck combined with flexion and rotation of the hip. Then, loose body removal and synovectomy were performed under the arthroscope (Figures 3 and 4). Most loose bodies could be removed using a probe or grasper, and smaller loose bodies could be removed by the arthroscopic suction technique according to their type and size. Intraoperative fluoroscopy could confirm the resection of big bodies. However, the size of loss bodies varies greatly in some cases (Figure 5); arthroscopy should be used to carefully check the various parts of the peripheral compartment and confirm a relatively thorough resection of loss bodies before closing.

### Postoperative Management

In order to prevent soft tissue adhesions, a tolerable range of motion (ROM) of hips was encouraged immediately after surgery. A passive ROM was first restored, followed by an active range and, finally, strength. Partial weight bearing was allowed 2 days after surgery, and full weight bearing was allowed 1 month after surgery.

### Clinical Evaluations

Clinical evaluations were performed both preoperatively and postoperatively. These evaluations comprised a detailed physical examination, the Harris hip score (HHS), hip ROM, and the visual analog scale (VAS) score.

### Statistical Analysis

Statistical analyses were conducted using the statistical program SPSS 12.0 (SPSS Inc., Illinois, USA). The means and the standard deviations of the results of individual factors, including the VAS scores, hip ROM, and HHSs before and after surgery, were calculated and statistically analyzed to identify any significant differences. The preoperative scores and last follow‐up scores were compared with a paired‐sample *t* test. *p* < 0.05 was considered indicative of statistical significance.

## Results

### Patients

Five patients (three male and two female) with an average age of 41 years (range 28–54 years) were included in this study. The mean follow‐up time was 25.2 months, ranging from 18 to 36 months postsurgery. Four patients had hip synovial osteochondromatosis on the right side, and one patient had it on the left side. Before surgery, all patients had dull hip pain, swelling, and restricted ROM. Baseline information of each patient is presented in Table 1.

**TABLE 1:** Baseline information of included cases.

Patient no.	Gender	Age	Side	Follow‐up period (months)
PT‐1	Male	54	Left	30
PT‐2	Male	41	Right	18
PT‐3	Female	40	Right	18
PT‐4	Male	42	Right	36
PT‐5	Female	28	Right	24

### Clinical Outcomes

Pre‐ and postoperative ROM (at the last follow‐up) is summarized in Table 2. Significant improvement was observed, except for abduction (*p* = 0.095). The VAS scores decreased from 3.2 ± 0.8 (range 2–4) to 0.4 ± 0.5 (range 0–1) (*p* < 0.001). The HHS improved from 58.6 ± 12.7 (range 43–73) preoperatively to 89.8 ± 5.3 (range 81–95) postoperatively at the final follow‐up (*p* < 0.001).

**TABLE 2:** Preoperative and postoperative ROM (degrees) in patients treated for hip synovial osteochondromatosis.

	Preoperative mean ± SD	Last follow‐up mean ± SD	*p*‐value
Flexion	92 ± 14.4	121 ± 7.4	0.003
Extension	11 ± 4.2	20 ± 3.5	0.0004
Abduction	35 ± 5	40 ± 3.5	0.095
Adduction	14 ± 8.2	22 ± 5.7	0.008
Internal rotation	10 ± 3.5	19 ± 4.2	0.018
External rotation	29 ± 7.4	40 ± 3.5	0.015

No major complications, such as perineal swelling, numbness, neurovascular injury, infection, thromboembolism, or severe persistent pain, were observed during the follow‐up period. Only one patient had asymptomatic hip synovial osteochondromatosis recurrence detected by regular x‐ray examination during a 12‐month follow‐up without any hip pain or clocking and did not require a revision arthroscopy.

## Discussion

A surgical technique of outside‐in hip arthroscopy without traction in the treatment of hip synovial osteochondromatosis was presented. The investigation of the medical records of those cases unveiled the notable clinical outcome of this technique with minimal complications.

### Unnecessity of Dislocation and Traction

Loose bodies are formed from metaplastic foci, and the calcification of these bodies is called synovial osteochondromatosis.^18^ The surgical removal of loose bodies is an optimal treatment for hip synovial osteochondromatosis. However, the necessity of hip dislocation or traction in treating hip synovial osteochondromatosis remains controversial. In open surgery, hip dislocation is necessary for the extensive debridement of loose bodies and proliferative synovial tissue, which is associated with the development of osteonecrosis of the femoral head and lesser trochanter fracture. In contrast, open hip surgery without hip dislocation can provide a good outcome, including clinical scores, patient satisfaction scores, and radiographic grades of osteoarthritis.^19^, ^20^

Loose bodies tend to accumulate in the peripheral area and are easily missed in traction. Therefore, traction in arthroscopic surgery may not be necessary for treating hip synovial osteochondromatosis and dislocation in open surgery. Previous studies showed that the classic arthroscopic approach for this condition was the inside‐out approach for capsulotomy. This method involved accessing the central compartment first under fluoroscopy, requiring joint traction. Although traction was necessary for complete diagnostic hip arthroscopy, it was associated with potential complications such as neurovascular injury or iatrogenic damage to the labrum or articular cartilage.^21^, ^22^

### Outside‐In Hip Arthroscopy Achieves Proper Visualization

In this study, we applied the extracapsular outside‐in approach to examine the peripheral compartment of the hip joint without traction, a technique developed at the end of the 20th century.^23^, ^24^ This approach creates a virtual space anterior to the hip capsule, and capsulotomy is performed to access the peripheral compartment,^17^ similar to a mini‐open anterior Hueter approach but conducted under endoscopic visualization.

A capsulotomy is usually needed for proper visualization of the joint. Synovial membranes and loose bodies located in the anterior and medial portions can be effectively removed. However, removing loose bodies located in the posteroinferior portion of the joint is difficult because the arthroscope cannot reach them easily. Therefore, we made an additional portal to slide the arthroscope under the femoral neck into the posteroinferior portion of the peripheral compartment and remove loose bodies located in this portion. At the same time, the hip was held in the position of flexion at 60° and external rotation at 20° by the assistant. As no intraoperative traction was applied, the hip position could be adjusted easily when we needed to increase the visual field, which is one of the advantages of the technique. In this study, the postoperative MRI revealed that most loose bodies were removed.

The retrieval of loose bodies and synovectomy were performed successfully and easily without traction. The results showed significant improvements in VAS scores, HHS, and ROM postsurgery. Also, no traction‐related complications, such as perineal numbness and swelling, were observed.

### Limitation of Arthroscopy Without Traction

A complete diagnostic hip arthroscopy requires traction, especially for a comprehensive inspection of the direct weight‐bearing cartilage, acetabular fossa, and ligamentum teres. Therefore, arthroscopy without traction was not effective in diagnosing and treating hip central compartment diseases such as FAI, acetabular cartilage injury, and loose bodies in the central compartment. In this study, the participants for surgery were selected in strict accordance with the established standards. MRI was performed on all patients before surgery to exclude those with any in the central compartment lesions. However, one patient in our study still had an asymptomatic recurrence of hip synovial osteochondromatosis and loose bodies in the acetabular fossa.

## Conclusion

Outside‐in hip arthroscopy without traction for treating hip synovial osteochondromatosis is a valid and effective option. It provides good clinical results, high patient satisfaction, rapid return to daily life, a short rehabilitation period, and few complications. Considering the limitation of accessing the central compartment without traction, proper patient selection is essential to exclude concomitant diseases, such as FAI, cartilage injury, and loose bodies in the central compartment.

## Author Contributions

This study was designed by Weigang Wu, Meng Liu, and Chiyuan Ma. Weigang Wu, Meng Liu, and Chiyuan Ma performed the surgeries. All patients' data were reviewed and analyzed by Huajie Mao, Chenhe Zhou, and Zhiqiang Wu. Figures were prepared by Weigang Wu and Huiguo Wu. Chiyuan Ma wrote the paper. All authors gave consent for publication.

## Conflict of Interest

The authors declare no conflicts of interest.

## Ethics statement

This study was approved by the Ethics Committee of the Second Affiliated Hospital, Zhejiang University School of Medicine, Hangzhou, China.
